# Clinical and Mechanistic Association Between Intestinal Permeability and the Gut Microbiome in Cirrhosis: Role of *Phascolarctobacterium*

**DOI:** 10.1002/ueg2.70262

**Published:** 2026-07-13

**Authors:** Rosa Haller, Nicole Feldbacher, Stefan Fürst, Johannes Woltsche, Lukas Gulden, Jakob Schwarzl, Julia Traub, Tobias Madl, Hansjörg Habisch, Angela Horvath, Vanessa Stadlbauer

**Affiliations:** ^1^ Division of Gastroenterology and Hepatology Department of Internal Medicine Medical University of Graz Graz Austria; ^2^ Center for Biomarker Research in Medicine (CBmed) Graz Austria; ^3^ Department of Clinical Medical Nutrition University Hospital Graz Graz Austria; ^4^ Otto Loewi Research Center Medicinal Chemistry Medical University of Graz Graz Austria; ^5^ BioTechMed‐Graz Graz Austria

**Keywords:** gut‐liver axis, gut metabolome, intestinal barrier, succinate, zonulin

## Abstract

**Background:**

In patients with liver cirrhosis, intestinal permeability and the composition of the gut microbiome are altered. Thus, the microbiome might be a therapeutic target for the treatment of both liver diseases and intestinal permeability. We aimed to investigate the relationship between the intestinal barrier and microbiome composition in cirrhosis and elucidate potential mechanisms for how bacteria influence permeability.

**Methods:**

We analyzed the fecal permeability biomarker zonulin by ELISA and microbiome composition by 16s rDNA sequencing in a discovery (*n* = 78) and a validation cohort (*n* = 90) of patients with liver cirrhosis. In the validation cohort, we analyzed the composition of the fecal metabolome by NMR spectroscopy. For mechanistic exploration, an intestinal barrier cell culture model using T84 cells was used.

**Results:**

In the discovery cohort (*n* = 78, 77% Child‐Pugh Grade A, 21% Child‐Pugh Grade B, 3% Child‐Pugh Grade C), decreasing zonulin levels in stool over 6 months were associated with higher *Phascolarctobacterium* abundance in the microbiome. *Phascolarctobacterium* was associated with better liver function (lower bilirubin, *p* = 0.04, INR *p* = 0.04, MELD Score, *p* = 0.02). Lower *Phascolarctobacterium* levels were observed in decompensated cirrhosis and were associated with 36‐month mortality in two cohorts. Metabolomics analysis showed an association between *Phascolarctobacterium* and lower succinate levels. Succinate increased gut permeability in vitro, and *Phascolarctobacterium succinatutens* strains improved the intestinal permeability, potentially by alleviating the effect of succinate.

**Conclusion:**

*Phascolarctobacterium* may represent a candidate biomarker of adverse outcomes in cirrhosis and a promising target for further investigation as a next‐generation probiotic involved in gut barrier function and succinate homeostasis.

**Trial Registration:**

NCT01607528, NCT03080129

## Key Summary

- Established knowledge on this subject:
  ◦ In patients with liver cirrhosis, the gut microbiome is altered and contributes to increased intestinal permeability, thereby raising the risk for complications and mortality, while also representing a potential therapeutic target
- What are the significant and/or new findings of this study?
  ◦ Lower *Phascolarctobacterium* abundance may be linked to more advanced or decompensated liver cirrhosis
  ◦ Higher *Phascolarctobacterium* is associated with better intestinal permeability and better outcome in liver cirrhosis.
  ◦ Our findings suggest a link between *Phascolarctobacterium*, succinate homeostasis, and gut barrier function in an in vitro cell culture model
  ◦ These findings support further investigation of *Phascolarctobacterium* as a potential biomarker and next‐generation probiotic for modulation of the intestinal barrier in liver cirrhosis.

## 1 Introduction

Liver cirrhosis is the 11th leading cause of death worldwide, accounting for up to one million deaths annually, often due to complications connected to disturbances of the gut–liver axis [1, 2]. The intestinal barrier is a dynamic system that continuously renews to preserve integrity and protect the host from external stressors [3]. In cirrhosis, this barrier is severely impaired, facilitating the translocation of bacterial products, which promote inflammation [4], infections [5], and further disease progression [4].

The gut microbiome is a key component of the intestinal barrier and plays an essential role in maintaining barrier function. In patients with cirrhosis, the microbiome is markedly altered [6], with an increase in pathogenic bacteria and a decrease in beneficial commensals [7]. These shifts not only enhance bacterial translocation but also reduce the production of protective short‐chain fatty acids (SCFAs), further compromising barrier integrity [7].

Although the gut barrier and microbiome have been proposed as sources of biomarkers and therapeutic targets, clinical translation remains limited because the dynamics and mechanistic links between barrier dysfunction and microbial alterations in cirrhosis are poorly understood [8]. We therefore aimed to characterize intestinal barrier dysfunction in cirrhosis using the direct permeability biomarker zonulin [9], with higher levels reflecting increased intestinal permeability [10], relate its changes to microbiome composition, and investigate potential causal relationships in vitro to identify novel biomarkers and therapeutic targets.

## 2 Methods

### 2.1 Study Protocol

#### 2.1.1 Discovery Cohort

Between July 2012 and September 2013, patients with liver cirrhosis were recruited at the outpatient clinic at the Department of Gastroenterology and Hepatology or the Department of Transplantation Surgery, both University Hospital Graz. The study was registered at clinicaltrials.gov (NCT01607528), approved by the research ethics committee of the Medical University of Graz (23–096 ex 10/11) and performed according to the Declaration of Helsinki. Eligible were men and women aged between 18 and 80 years. Liver cirrhosis diagnosis was based on liver histology or characteristic clinical and radiological features. Exclusion criteria were Child‐Pugh score ≥ 12, alcohol abuse within 2 weeks before inclusion, active infection at screening, antibiotic therapy, excluding permanent prophylaxis, intake of pro‐/pre‐/synbiotics within 2 weeks, gastrointestinal haemorrhage within 2 weeks before inclusion, immunomodulatory drugs, hepatic encephalopathy of stage two or higher, renal failure (creatinine > 1.7 mg/dL), pancreatitis, other severe diseases unrelated to cirrhosis, malignancy, suspected noncompliance and pregnancy. Patient outcome was assessed after 36 months. The entire patient cohort (*n* = 101) was previously described in more detail [11].

#### 2.1.2 Validation Cohort

Hospitalized patients with liver cirrhosis were recruited at the Medical University of Graz between April 2017 and January 2019. The study was registered at clinicaltrials.gov (NCT03080129) and was approved by the research ethics committee of the Medical University of Graz (29–280 ex 16/17). It was conducted after informed consent according to the principles of the Declaration of Helsinki. Men and women over 18 years who gave written consent and with a diagnosis of liver cirrhosis (clinical/radiological and computer tomography/magnetic resonance imaging). Patients with hepatic encephalopathy > grade 2, other cognitive disorders or conditions precluding informed consent, hepatocellular carcinoma (Barcelona Clinic Liver Cancer stage C/D), ursodeoxycholic acid treatment and intake of probiotics and antibiotics (except for long term prophylaxis) at the study visit were excluded. A single study visit was performed per patient. Patient outcome was assessed after 36 months. The entire patient cohort (*n* = 178) was previously described in more detail [12]; in this study, only patients with liver cirrhosis (*n* = 98) were included.

### 2.2 Gut Permeability Measurement

Markers for gut permeability were measured using commercially available enzyme‐linked immunosorbent assays (ELISA). Zonulin (K5600, Immundiagnostik AG, Bensheim, Germany) and Calprotectin (K6927, Immundiagnostik AG, Bensheim, Germany) were measured in stool. LBP (HK315, Hycult Biotechnology, Uden, Netherlands), sCD14 (DC140, R and D Systems, Abingdon, UK), and diamino‐oxidase (DAO) (K8500, Immundiagnostik AG, Bensheim, Germany) were measured in serum.

### 2.3 Gut Microbiome Analysis

The gut microbiome was analyzed by 16S rDNA sequencing. DNA was isolated from the stool samples with the MagNA Pure LC DNA Isolation Kit (Roche, Mannheim, Germany). Hypervariable region V1‐V2 was amplified (*discovery cohort*; forward primer: AGAGTTTGATCCTGGCTCAG; reverse primer: CTGCTGCCTYCCGTA, conducted by the Core Facility Molecular Biology of the Medical University of Graz in 2013; *validation cohort*; forward primer: AGAGTTTGATCCTGGCTCAG, reverse primer: TGCTGCCTCCCGTAGGAGT, conducted by the Institute of Clinical Molecular Biology Kiel in 2020) and sequenced using Illumina Miseq technology (Illumina, Eindhoven, The Netherlands) [13]. Sequence reads were pre‐processed on QIIME2 on a local Galaxy server (https://galaxy.medunigraz.at). Denoising was performed with the Divisive Amplicon Denoising Algorithm (DADA2). Taxonomy was assigned based on the Silva V132 database with a Naïve Bayes classifier. The OTU table obtained, including taxonomic information, was imported into *R* (version 4.3.3) for further analysis. Cyanobacteria were excluded from the analysis. Sequencing data are available in the NCBI Sequencing Read Archive *(discovery cohort*: SRP132827, https://www.ncbi.nlm.nih.gov/sra/SRP132827 *, validation cohort*: PRJNA933898, https://www.ncbi.nlm.nih.gov/sra/PRJNA933898).

In the discovery cohort, analysis was performed for all patients with available baseline gut microbiome composition and zonulin dynamics data (*n* = 75). Alpha diversity was observed by Chao1, Shannon, Inverse Simpson, and Richness. Beta diversity was analyzed with Bray‐Curtis. Analysis of Composition of Microbiomes (ANCOM) and Linear discriminant analysis Effect Size (LefSe) were performed. Microbiome Multivariable Associations with Linear Models (MaAsLin 3, in *R* version 4.5.2) was performed with including Child Pugh Score to assess disease severity. Key taxa were analyzed for their relationship with clinical markers. In the validation cohort, patients with liver cirrhosis (*n* = 98) were analyzed. Patients with < 14,397 sequence reads (minimal sequence reads of the discovery cohort) were excluded from the analysis (*n* = 8). Key taxa analyzed for validation from the remaining validation cohort (*n* = 90).

### 2.4 NMR Metabolomics Analysis

Stool samples were prepared as described previously [14]. Shortly, samples were combined with 66% methanol/water. After homogenization using zirconium oxide beads and centrifugation, the supernatant was lyophilized and resuspended in 0.08 mM sodium phosphate buffer in D_2_O, pH 7.4 with 4.6 mM trimethylsilyl propionic acid (d_6_‐TMSP) and quantified at 310 K by NMR spectroscopy (Bruker Avance 600 MHz). The acquired spectra were aligned and normalized by probabilistic quotient normalization. Finally, metabolite integrals were analyzed in MetaboAnalyst 6.0 (www.metaboanalyst.ca, 15.01.2025). The stool metabolome of 83 patients from the validation cohort was normalized by sum, log10 transformed, mean‐centered, and divided by the standard deviation of each variable. Differences between groups were assessed with orthogonal Partial Least‐Squares Discriminant Analysis (orthoPLS‐DA); metabolites with a variable of importance (VIP) score ≥ 1 were considered significant [15].

### 2.5 Cell Cultivation

T84 cells, a colorectal cancer cell line obtained from a lung metastasis, (88,021,101, ECACC, Porton Down, United Kingdom) [16] were cultivated in a medium containing a 1:1 mixture of Dulbecco's Modified Eagle's Medium (DMEM) (41,965,062, Gibo, ThermoFisher Scientific, Waltham, MA, USA) and Ham's F‐12 nutrient mix (N6658, Sigma‐Aldrich, Merck KGaA, Darmstadt, Germany) supplemented with 5% fibrinogen depleted pooled human platelet lysates (pHPL) (UBT Graz [17], fibrinogen depletion described in the supplementary) and 1% Penicillin‐Streptomycin (P4333, Sigma‐Aldrich, Merck KGaA, Darmstadt, Germany). The cells were seeded in 75 cm^2^ polystyrene tissue culture flasks (CLS430641U, Corning, NY, USA) and cultivated in a humidified incubator at 37°C with 5% CO_2_.

### 2.6 Bacteria Cultivation

*Phascolarctobacterium succinatutens* strains A177 (DSM22533, DSMZ, Leibniz, German) and B162 (DSM 22521, DSMZ, Leibniz, German) were cultivated for 20 h in DSMZ Medium 104c with 8 g/L sodium succinate (Supporting Information S1: Table S1) in anoxic conditions (80% Nitrogen, 20% CO_2_) before the experiment.

### 2.7 Intestinal Barrier Model

For intestinal barrier experiments, 1 × 10^4^ T84 were seeded into 0.33 cm^2^ polystyrene transwell permeable support cell culture inserts (0.4 µm pore size) (CLS3470, Corning, NY, USA) and were grown in media without antibiotics. Cells were cultivated until they formed a monolayer (> 1000 Ω*cm^2^). Transepithelial electrical resistance (TEER) was measured with EVOM Manual Epithelial Volt Ω Meter (EVM‐MT‐03–01, EVM‐EL‐03–03‐01, World Precision Instruments, Sarasota, FL, USA) and calculated from resistance (R) and surface (A): TEER = (R – R_Background_) * A. For the experiment, the apical cell medium was replaced with the respective conditions, and the cells were treated for 17 h. All conditions were measured as duplicates. TEER was measured once shortly before and at the end of the treatment. The difference between the baseline TEER and final TEER was analyzed. Every experiment was conducted three times. Sodium Succinate was dissolved to 1M in T84 growth medium to avoid pH changes. The stock solution was diluted to final concentrations of 200, 170, 160, 150, 140, 130, 120, 110, 100, and 50 mM. *P. succinatutens* strains were cultivated as described. Before the experiment, the culture was centrifuged at 500g for 10 min. The supernatant was decanted, and the pellet was dissolved in T84 growth medium with or without succinate and applied to the respective wells. Experiments were analyzed and visualized in *R* (version 4.3.3).

### 2.8 Statistical Analysis

Bacteria were categorized into high and low abundance groups based on the area under the receiver operating characteristic curve (AUROC) and the Youden index based on mortality. Survival analysis was conducted using Kaplan‐Meier curves. The log‐rank test was applied to assess the statistical significance. Cox‐Regression was performed to determine the hazard‐ratio (HR) and 95% confidence interval (CI). If one group had low or few events, Firth's penalized maximum likelihood bias reduction [18] was used. Multivariable Cox regression analysis was performed with adjustment for Child–Pugh score. For the prediction of new decompensation for 12 months, logistic regression was performed. In cases of sparse data or low event counts, Firth's bias‐reduced logistic regression was applied [19]. For this analysis, patients from both cohorts were stratified into Child–Pugh grade A/B (*n* = 152) and Child–Pugh grade C (*n* = 13). The Child A/B group was further subdivided according to baseline decompensation status (*n* = 57). Depending on the data distribution, comparative analyses were performed using either Mann‐Whitney *U* test, Student's t‐test, Wilcoxon test, or paired *t*‐test. Normality was assessed using Shapiro‐Wilk test. Categorical variables were tested using the chi‐square test, Fisher's exact, McNemar or Stuart‐Maxwell test. *p*‐values were adjusted using Benjamini‐Hochberg (BH) correction for respective groups (indicated in the tables). Statistical analysis was performed in *R* (version 4.3.3). All used packages are cited in the supplements.

## 3 Results

### 3.1 Patient Characteristics

Seventy‐eight patients of the discovery cohort and 90 patients of the validation cohort were included in the analysis. The demographic data and gut barrier markers are shown in Table 1. Antibiotic prophylaxis at sampling was taken by 1 patient in the discovery cohort included (norfloxacin), and 7 patients in the validation cohort (rifaximin (*n* = 6) and rifampicin (*n* = 1)).

**TABLE 1:** Demographic characteristics and gut barrier marker in both study cohorts.

	Discovery cohort (*n* = 78)	Validation cohort (*n* = 90)
General characteristics		
Deceased after 36 months	8 (10%)	38 (42%)
Age [years]	58 (55; 60)	64 (62; 67)
BMI [kg/m^2^]	27 (25.4; 27.68)	27.29 (25.7; 28.4)
Bilirubin [mg/dl]	1.3 (1; 1.5)	1.2 (1; 1.4)
Albumin [g/dl]	4.2 (4; 4.4)	3.5 (3.4; 3.6)
INR	1.2 (1.2; 1.3)	1.2 (1.2; 1.3)
KREA [mg/dl]	0.8 (0.8; 0.9)	0.9 (0.8; 1)
CRP [mg/l]	2.4 (1.5; 2.9)	5.6 (3.4; 7.5)
MELD score	10 (9; 12)	10 (9; 11)
PPI Intake	44 (56%)	47 (52%)
Beta‐blocker intake	42 (54%)	56 (62%)
Men	56 (72%)	67 (74%)
Child‐Pugh grade (A/B/C)	60 (77%)/16 (21%)/2 (3%)	42 (47%)/36 (40%)/12 (13%)
Antibiotic prophylaxis	1 (1%)	7 (8%)
Diabetes	24 (31%)	38 (42%)
Etiology (ALD/HCV/other)	42 (54%)/13 (17%)/23 (29%)	53 (59%)/15 (17%)/22 (24%)
Gut‐liver axis biomarker		
Zonulin [ng/mg]	78.1 (69.9; 84.4)	98.7 (86.2; 110.7)
LBP [μg/ml]	18.5 (15.7; 20.9)	19 (16.8; 21.6)
Calprotectin [μg/g]	114.7 (59.2; 180.1)	84.7 (64.4; 121.7)
sCD14 [ng/ml]	1.9 (1.7; 2.07)	1.8 (1.7; 1.8)
DAO [U/ml]	18 (16; 20)	18.9 (15.8; 23.2)

*Note:* The median and the 95% CI are given for continuous variables.

Abbreviations: ALD, Alcoholic Liver Disease; BMI, Body Mass Index; CRP, C‐reactive protein; DAO, Diamine oxidase; HCV, Hepatitis C; INR, Internation Normalized Ratio; LBP, Lipopolysaccharide Binding Protein; MELD, Model of End Stage Liver Disease; PPI, Proton Pump Inhibitors; sCD14, soluble Cluster of Differentiation 14.

The clinical characteristics, liver function, drug intake, and markers of intestinal barrier function were comparable between the two cohorts, but differences could be observed in age and albumin levels. Mortality and Child‐Pugh Grade were higher in the validation cohort.

### 3.2 Zonulin Changes Were Associated With Other Gut‐Liver Markers

Baseline zonulin levels, as a marker of gut permeability, were not associated with mortality in the discovery cohort (Supporting Information S1: Figure S1). Given the dynamic nature of the gut barrier, we assessed zonulin dynamics over 6 months. We defined decreasing zonulin levels as improving and unchanged or increasing zonulin levels as deteriorating (Table 2). Zonulin (*p* = 0.00008) and lipopolysaccharide‐binding protein (LBP; *p* = 0.0088) at baseline were significantly higher in patients with improved zonulin levels. At the same time, DAO, another marker of intestinal permeability, was significantly lower at baseline in patients with improved zonulin levels (*p* = 0.011). LBP levels decreased in parallel with zonulin levels after 6 months. In patients with deteriorating zonulin, calprotectin levels increased significantly after 6 months (*p* = 0.02), whereas they were not different at baseline. The two groups did not differ in other characteristics, drug intake, or liver function at baseline. Beta blocker intake, PPI intake, alcohol cessation rate within six months or number of decompensation events and infections within 12 months were not different between the groups (Supporting Information S1: Table S2). There was no TIPS placement or new introduction of rifaximin within 6 months. Therefore, these factors were unlikely to account for the observed changes in zonulin levels.

**TABLE 2:** Patient's characteristics of the discovery cohort depending on changes in zonulin levels at baseline and after 6 months.

	Discovery Cohort							
	Improving zonulin (*n* = 33)			Deteriorating zonulin (*n* = 45)			*p*‐adj	
Age [years]	56 (52; 62)			59 (55; 61)			0.44	
Etiology (ALD/HCV/other)	15 (46%)/6 (18%)/12 (36%)			27 (60%)/7 (16%)/11 (24%)			0.52	
Diabetes	7 (21%)			17 (38%)			0.44	
Men	21 (64%)			35 (78%)			0.44	
Antibiotic prophylaxis	0 (0%)			1 (2%)			> 0.99	
	**Baseline**	**Six months**	***p*‐adj**	**Baseline**	**Six months**	***p*‐adj**	**Base‐line**	**Six months**
BMI [kg/m^2^]	**25.6 (24.3; 29.4)**	**26.6 (24.6; 29.4)**	**0.04**	27.1 (26.5; 28.1)	26.9 (25.8; 28)	> 0.99	> 0.99	0.90
Alcohol use	7 (21%)	5 (15%)	0.75	10 (22%)	9 (20%)	> 0.99	> 0.99	0.90
PPI intake	18 (55%)	17 (52%)	> 0.99	26 (58%)	25 (56%)	> 0.99	> 0.99	0.90
Beta‐blocker intake	17 (52%)	19 (58%)	0.73	25 (56%)	28 (62%)	> 0.99	> 0.99	0.90
Bilirubin [mg/dl]	1 (0.7; 1.4)	0.9 (0.7; 1.3)	0.22	1.3 (1; 1.7)	1.3 (1; 1.9)	> 0.99	0.24	0.13
Albumin [g/dl]	4.3 (4; 4.6)	4.2 (3.9; 4.4)	0.22	4.2 (3.9; 4.4)	4.2 (4; 4.3)	> 0.99	0.21	0.75
INR	1.2 (1.1; 1.3)	1.2 (1.1; 1.3)	0.22	1.3 (1.2; 1.4)	1.3 (1.2; 1.4)	> 0.99	0.12	0.90
Creatinine [mg/dl]	0.8 (0.8; 0.9)	0.8 (0.8; 0.9)	0.57	0.8 (0.8; 0.9)	0.8 (0.8; 0.9)	> 0.99	> 0.99	0.90
CRP [mg/l]	2.6 (1.2; 3.5)	2.8 (1.9; 5.1)	0.22	2.2 (1.4; 2.9)	2 (1.3; 2.5)	> 0.99	> 0.99	0.68
MELD score	9 (8; 11)	9 (8; 11)	0.73	11 (10; 13)	11 (10; 14)	> 0.99	0.18	0.13
Child‐Pugh grade (A/B/C)	29 (88%)/4 (12%)	30 (90%)/3 (9%)	> 0.99	31 (69%)/12 (27%)/2 (4%)	31 (69%)/11 (24%)/3 (7%)	> 0.99	0.26	0.15
Gut‐liver axis biomarker								
Zonulin [ng/mg]	**93.1 (78.5; 100.4)**	**71.8 (58; 80.6)**	**1.164e‐09**	**64.9 (56.9; 78.8)**	**82.2 (71.4; 95)**	**1.258500e‐07**	**0.0004**	**0.02**
LBP [μg/ml]	**26.1 (19.5; 33.8)**	**19.2 (16.5; 23)**	**0.04**	**16.7 (13.4; 18.9)**	16.6 (15; 18.9)	> 0.99	**0.009**	0.12
Calprotectin [μg/g]	141.5 (47.7; 263.6)	161 (46.5; 297.7)	0.79	**95.3 (43.5; 180.1)**	**151.2 (88.2; 206.3)**	**0.02**	0.26	0.64
sCD14 [ng/ml]	1.8 (1.7; 2.3)	2.2 (1.7; 2.3)	0.79	1.9 (1.7; 2.1)	1.9 (1.7; 2)	> 0.99	0.65	0.15
DAO [U/ml]	**15.5 (13; 18)**	15.5 (13.5; 18)	0.79	**21.3 (18; 25)**	18.71 (16; 21.79)	0.64	**0.01**	0.10

*Note:* The median and the 95% CI are given for continuous variables. Significantly altered variables are marked in bold (*p* < 0.05).

Abbreviations: BMI, Body Mass Index; CRP,C‐reactive protein; DAO, Diamine oxidase, HCV, Hepatitis C; INR, Internation Normalized Ratio; LBP, Lipopolysaccharide Binding Protein; MELD, Model of End Stage Liver Disease; *p*‐adj – adjusted *p*‐value. PPI, Proton Pump Inhibitors; sCD14, soluble Cluster of Differentiation 14.

### 3.3 Distinct Changes in Microbiome Composition Between Patients With Improving and Deteriorating Zonulin Levels

**FIGURE 1:**
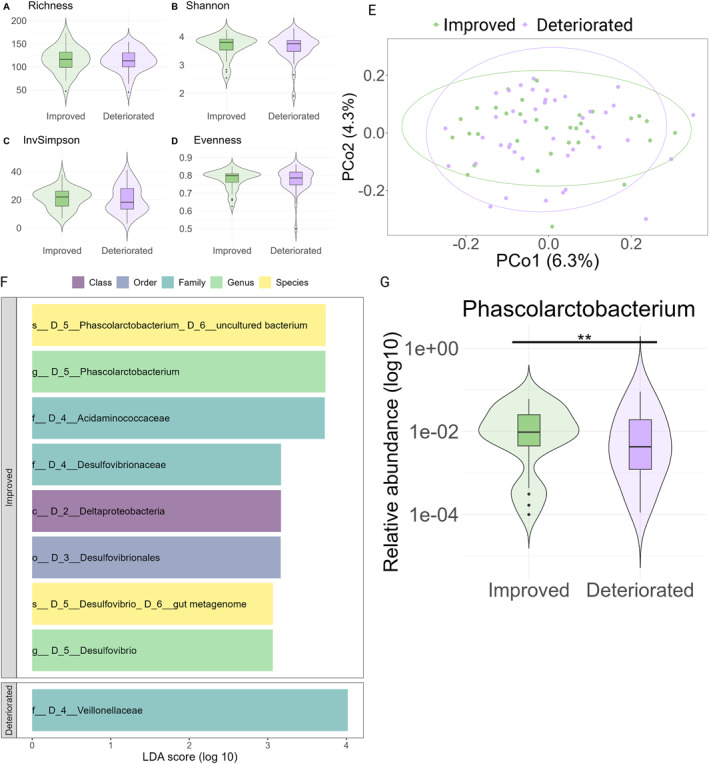
Alpha‐diversity was unchanged depending on gut barrier changes (A–D). Beta‐diversity was not altered depending on the gut barrier (E). LefSe analysis showed several distinct changes (F). ANCOM identified *Phascolarctobacterium* as an altered taxa (G). All panels refer to the discovery cohort. LDA – Linear Discriminant Analysis.

There was no difference between alpha‐ and beta diversity at baseline between patients with improving and deteriorating zonulin levels in the discovery cohort (Figure 1A–E). Taxonomically LefSe analysis identified several biomarkers; the *Veillonellacea* family was associated with deteriorating zonulin levels; Acidaminococcaceae*, Phascolarctobacterium* uncultured bacterium, *Phascolarctobacterium,* Desulfovibrionaceae*, Desulfovibrionales*, *Deltaproteobacteria*, *Desulfovibrio,* and *Desulfovibrio* gut metagenome were associated with improving zonulin levels (Figure 1F). ANCOM analysis validated that a higher abundance of *Phascolarctobacterium* uncultured bacterium and *Phascolarctobacterium* were associated with improving zonulin levels (*p* = 0.0021) (Figure 1G). MaAsLin three identified no significant genera (Supporting Information S1: Figure S2).

### 3.4 *Phascolarctobacterium* Was Associated With a Better Prognosis

**FIGURE 2:**
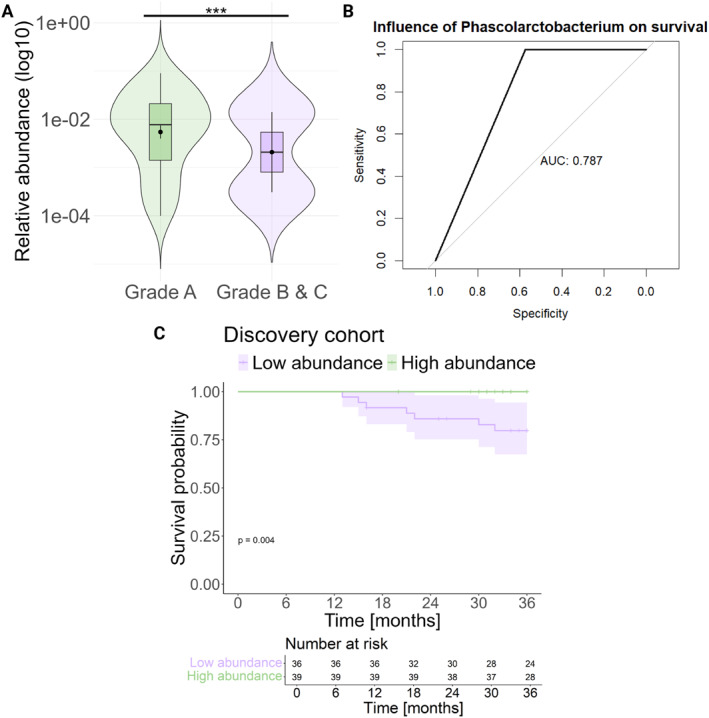
*Phascolarctobacterium* abundance (log10 transformed) is significantly higher in patients with Child‐Pugh Grade A (A). AUROC analysis of *Phascolarctobacterium* abundance and mortality after 36 months (B). Patients with higher *Phascolarctobacterium* abundance have increased survival chances over 36 months (C). All panels refer to the discovery cohort. ***p < 0.001.

The relationship between *Phascolarctobacterium* and zonulin levels led us to further investigate *Phascolarctobacterium* in the discovery cohort. *Phascolarctobacterium* abundance was significantly higher in patients with Child‐Pugh Grade A compared to those with Grade B and C (*p* = 0.0004) in the discovery cohort (Figure 2A).

Therefore, we tested the predictive power of *Phascolarctobacterium* for 36‐month mortality. The AUROC analysis demonstrated that *Phascolarctobacterium* could predict outcome (AUC = 0.787). The optimal cutoff of *Phascolarctobacterium* abundance was determined by the Youden index (cutoff = 5x10^−5^ relative abundance, specificity = 0.574, sensitivity = 1). Lower *Phascolarctobacterium* abundance was associated with an increased risk of mortality (*p* = 0.004, HR = 0.0562, 95% CI = 0.00043; 0.4613) (Figure 2B–C). After adjustment for the Child–Pugh score in multivariable Cox regression, this association remained an independent predictor (*p* = 0.045, HR = 0.0968, 95% CI = 0.00072–0.9569). Patients with low *Phascolarctobacterium* abundance also had worse liver function, as shown by significantly elevated bilirubin levels (*p* = 0.04), increased INR (*p* = 0.04), as well as MELD scores (*p* = 0.02) and Child‐Pugh Grade (*p* = 0.004) (Table 3).

**TABLE 3:** Patient characteristics at baseline from the discovery cohort and the validation cohort for low and high *Phascolarctobacterium* abundance.

	Discovery cohort		Validation cohort			
	Low abundance (*n* = 36)	High abundance (*n* = 39)	Low abundance (*n* = 69)		High abundance (*n* = 21)	
Deceased	7 (19%)	0 (0%)	35 (51%)		4 (19%)	
Demographic characteristics						
			***p*‐adj**			**p‐adj**
Age [years]	59 (54; 62)	57 (53; 63)	> 0.99	63 (61; 67)	69 (62; 74)	0.13
Etiology (ALD/HCV/Others)	21 (58%)/4 (11%)/11 (31%)	19 (49%)/8 (20%)/12 (31%)	0.83	41 (59%)/11 (16%)/17 (25%)	12 (57%)/4 (19%)/5 (24%)	0.98
Diabetes	12 (33%)	12 (31%)	> 0.99	28 (41%)	10 (48%)	0.79
PPI Intake	22 (61%)	19 (49%)	0.75	35 (51%)	12 (57%)	0.73
Beta‐blocker intake	18 (50%)	22 (56%)	> 0.99	41 (59%)	15 (71%)	0.69
Men	26 (72%)	27 (69%)	> 0.99	47 (68%)	20 (95%)	0.12
Antibiotic prophylaxis	1 (3%)	0 (0%)	> 0.99	7 (10%)	0 (0%)	0.40
BMI [kg/m^2^]	27.2 (25.9; 28.9)	25.4 (23.9; 27.8)	0.50	27.3 (25.3; 28.4)	27.1 (22.2; 29)	0.39
Bilirubin [mg/dl]	**1.5 (1.1; 2.3)**	**1 (0.7;1.3)**	**0.04**	1.3 (1; 1.9)	0.9 (0.6; 1.2)	0.12
Albumin [g/dl]	4.2 (3.5; 4.5)	4.2 (4.0; 4.5)	0.42	3.5 (3.3; 3.6)	3.7 (3.3; 3.9)	0.39
INR	**1.3 (1.2; 1.4)**	**1.2 (1.1; 1.3)**	**0.04**	1.2 (1.2; 1.3)	1.1 (1.2; 1.3)	0.13
KREA [mg/dl]	0.8 (0.8; 0.9)	0.8 (0.7; 0.9)	> 0.99	0.8 (0.8; 1)	1 (0.8; 1.2)	0.39
CRP [mg/l]	2.6 (1.5; 3.4)	2 (1.2; 3)	0.69	5 (3.0; 7.8)	6.2 (3.4; 26)	0.20
MELD score	**13 (10; 15)**	**9 (8; 10)**	**0.02**	11 (9; 12)	10 (8; 12)	0.39
Child‐pugh grade (A/B/C)	**21 (58%)/14 (39%)/1 (3%)**	**37 (95%)/2 (5%)**	**0.004**	29 (42%)/29 (42%)/11 (16%)	13 (62%)/7 (33%)/1 (5%)	0.39
Gut‐liver axis biomarker						
Zonulin [ng/mg]	72.5 (54.2; 83.1)	84.2 (69; 95.6)	0.29	102.2 (87.8; 126.6)	94.4 (62.4; 123)	0.25
LBP [μg/ml]	17.2 (14.55; 20.7)	20.7 (14.7; 27.9)	0.62	18 (4.9; 20.6)	21.9 (17; 26.8)	0.1
Calprotectin [μg/g]	98.2 (43.5; 205.7)	114.7 (41.7; 217.8)	0.88	81.5 (63; 121.7)	101.1 (36.2; 157.5)	0.71
sCD14 [ng/ml]	1.9 (1.7; 2.1)	1.9 (1.7; 2.1)	0.88	1.8 (1.7; 1.8)	2.1 (1.6; 2.2)	0.71
DAO [U/ml]	21.5 (15.5; 25.5)	16.5 (14.5; 19)	0.35	19.83 (16.6; 26.9)	13.66 (8.9; 23.1)	0.07

*Note:* The median and the 95% CI are given for continuous variables. Significantly altered variables are marked in bold (*p* < 0.05).

Abbreviations: BMI, Body Mass Index; CRP,C‐reactive protein; DAO, Diamine oxidase, HCV, Hepatitis C; INR, Internation Normalized Ratio; LBP, Lipopolysaccharide Binding Protein; MELD, Model of End Stage Liver Disease; *p*‐adj – adjusted *p*‐value. PPI, Proton Pump Inhibitors; sCD14, soluble Cluster of Differentiation 14.

**FIGURE 3:**
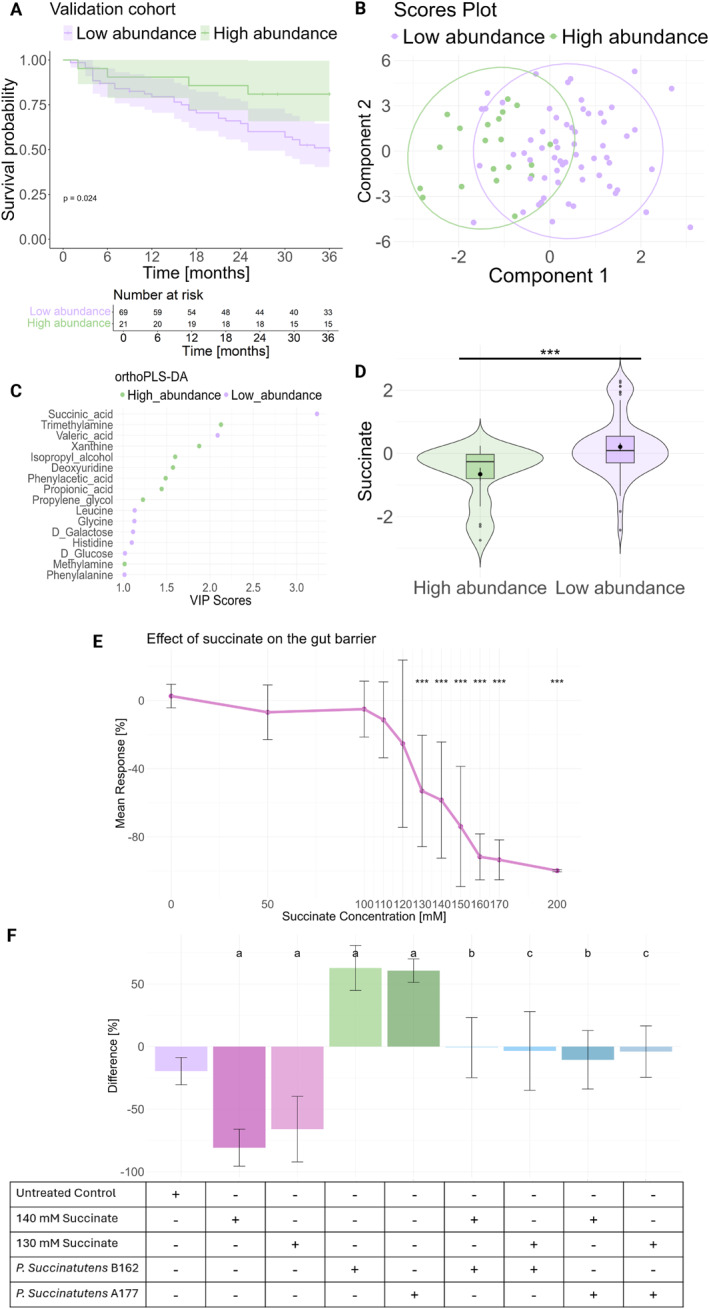
Patients with higher *Phascolarctobacterium* abundance have increased survival chances over 36 months (A). OrthoPLS‐DA score plot of the stool metabolome depending on *Phascolarctobacterium* abundance (B). Discriminatory metabolites identified by orthoPLS‐DA (all VIP ≥ 1) (C). Succinate is significantly increased in patients with low *Phascolarctobacterium* abundance (D). Influence of sodium succinate on an in vitro gut barrier model (***p < 0.001, compared to 0 mM) (E). Influence of sodium succinate and *P. succinatutens* B162 and A177 on the in vitro barrier model (a – significant to the control, b – significant to 140 mM sodium succinate, c – significant to 130 mM sodium succinate) (F). Panels A–D refer to the validation cohort.

The predictive power of *Phascolarctobacterium* for 36‐month mortality could be reproduced in a validation cohort (*p* = 0.024, HR = 0.323, 95% CI = 0.1145; 0.9109) (Figure 3A, Supporting Information S1: figure S3). Importantly, this association remained significant after adjustment for Child‐Pugh score in multivariable Cox regression (*p* = 0.0305, HR = 0.3165, 95% CI = 0.1116–0.8973), whereas Child‐Pugh score was not significantly associated with mortality (*p* = 0.7048). In the validation cohort, no differences in clinical characteristics, drug intake, liver function, or intestinal barrier markers were observed between patients with high and low *Phascolarctobacterium* abundance (Table 3).

In contrast, logistic regression analysis did not demonstrate a significant association between baseline *Phascolarctobacterium* abundance and 12‐month decompensation. In patients from both cohorts with Child A/B cirrhosis without baseline decompensation, no association was observed (OR = 0.87, *p* = 0.76) or in the patients with baseline decompensation (OR = 0.45, *p* = 0.18). In Child C patients, Firth's penalized logistic regression was applied, which also did not show a statistically significant association (OR = 0.18, *p* = 0.28).

### 3.5 Association Between *Phascolarctobacterium* and the Fecal Metabolome

We aimed to investigate the association between *Phascolarctobacterium* and fecal metabolites. To achieve this, we analyzed the stool metabolomes of 83 patients from the validation cohort. OrthoPLS‐DA scores showed differences between patients with high and low *Phascolarctobacterium* abundance (Figure 3B) and identified 16 significantly altered metabolites (VIP ≥ 1). The strongest difference was found for succinic acid (VIP = 3.23) (Figure 3C), the primary energy source of *Phascolarctobacterium*. Fecal succinate levels were significantly lower in patients with high *Phascolarctobacterium* abundance (*p* = 0.012) (Figure 3D).

### 3.6 Succinate Impairs Gut Barrier Function and *Phascolarctobacterium* Improves Gut Barrier Function

We hypothesized that *Phascolarctobacterium* positively affects the gut barrier by metabolizing succinate to propionate. First, we tested the effect of succinate in several concentrations on a gut barrier model in vitro. Treatment with sodium succinate reduced TEER in a concentration‐dependent manner. Concentrations higher than 150 mM significantly decreased TEER by 80%–100% (*p* = 0.004, Figure 3E) compared to the baseline measurement. For further experiments, 130 and 140 mM sodium succinate were chosen as they were the lowest concentrations that decreased TEER significantly (*p* = 0.004). *P. succinatutens* A177 and B162 alone increased TEER (*p* = 0.004). Combined treatment with sodium succinate and *P. succinatutens* led to a complete reversal of succinate‐induced impairment of the barrier function (Figure 3F, Supporting Information S1: Table S3).

## 4 Discussion

We assessed the role of the gut–liver axis, focusing on intestinal permeability and the gut microbiome to identify novel biomarkers and therapeutic targets. Our study demonstrated for the first time that dynamics in intestinal permeability, assessed by zonulin, were associated with alterations in microbiome composition. *Phascolarctobacterium*, a succinate metabolizing gut commensal [20], was linked with zonulin dynamics, liver function and mortality, but not acute decompensation. Furthermore, we showed that the succinate metabolizing capacity of *Phascolarctobacterium* may be causally related to improved barrier function in an in vitro model of the intestinal barrier.

We analyzed the changes in zonulin, a tight junction regulator [9], as a direct marker for gut barrier function. We previously showed that fecal zonulin levels were reduced with probiotic intervention, suggesting an influence of the gut microbiome on zonulin [13].

The changes in zonulin in our cohort were mirrored by changes in the other established and widely used markers of gut barrier function in both stool and serum gut‐liver axis markers [11, 12, 21]: LBP, DAO, and calprotectin. This panel was chosen to reflect barrier dysfunction (zonulin and DAO), intestinal inflammation (calprotectin) and bacterial translocation (LBP and sCD14). Like zonulin, LBP was higher at baseline in patients with improving zonulin levels and decreased over 6 months. LBP is an acute‐phase protein [22, 23]. It was suggested that repeated measurements of LBP enhance its predictive power for long‐term outcomes [24], aligning with our observation that LBP levels can improve over time. DAO levels were lower in patients with improved zonulin levels, indicating that DAO might be an earlier marker of gut health or measure a different mechanism of intestinal permeability. High DAO levels in patients with hepatitis B were associated with readmission [25] and DAO was reported to be elevated in patients undergoing orthotopic liver transplantation [26]. Calprotectin increased after 6 months in patients with deteriorating zonulin levels in our cohort, indicating increased intestinal inflammation. Calprotectin levels were previously found to be elevated in patients with liver disease [27], and single measurements were able to predict mortality or the need for liver transplantation [28], supporting the pathophysiological relevance of intestinal inflammation. Overall, our panel of gut‐liver axis biomarkers exhibit consistent and similar changes, suggesting that repeated measurements improve the power of gut liver‐axis biomarkers.

The BMI of patients with improved zonulin levels increased slightly, also after correcting for ascites as an indication for fluid overload, possibly indicating improved nutritional health status; as in cirrhosis, a low BMI can be a sign of malnutrition, increasing the risk for complications [29]. This may be caused by decreased inflammation through an improved intestinal barrier. The weight and height of patients, however, were self‐reported in our study and should be treated cautiously because of potential reporting bias. Interestingly, the improvement of the gut‐liver axis markers appears to be independent of liver and inflammation markers, suggesting other factors, such as the gut microbiome, may play an important role.

We investigated the relationship between intestinal barrier function and microbiome composition in cirrhosis. We were able to demonstrate an association between zonulin dynamics and taxonomic changes where a higher *Phascolarctobacterium* abundance was associated with improved zonulin levels. *Phascolarctobacterium* is generally considered a beneficial bacterium because it produces the SCFA propionate [20] via the succinate pathway [30]. Fecal succinate is predominantly produced by the gut microbiome [31]. A shift toward an increased abundance of succinate producers and a decrease in consumers in the gut is associated with diseases [32].

Decreased *Phascolarctobacterium* abundance has been reported in IBD [33] and *Clostridioides difficile* infections [34]. This may relate to its use of succinate as a sole energy source, a rare and low‐yield strategy [35] in the human gut, whereas other bacteria, including *C. difficile*, use succinate alongside additional substrates [34]. By consuming succinate, *Phascolarctobacterium* can limit the growth of opportunistic pathogens [34]. However, *Phascolarctobacterium* is associated with cognitive disorders [36, 37].

In cirrhosis, a low abundance of *Phascolarctobacterium* was associated with an increased risk of hepatic encephalopathy recurrence [38], and it was described to be lower in patients with Hepatitis B [39]. *Phascolarctobacterium* was indirectly associated with improved gut barrier function by producing propionate [30, 40]; our study is now the first to directly link *Phascolarctobacterium* abundance to improved gut barrier function in silico and in vitro. Notably, the observed barrier‐enhancing effect of *Phascolarctobacterium* in the absence of exogenously added succinate suggests that additional factors, such as residual substrate availability or alternative bacterial metabolites, may contribute to the modulation of epithelial barrier function. The exact underlying mechanism remains to be determined.

Succinate homeostasis in the human gut is important for the intestinal barrier and immune function [33, 41, 42]. Specific succinate‐consuming bacteria could therefore function as probiotics to restore succinate homeostasis [32]. We showed in vitro that succinate impairs the gut barrier, suggesting a potential mechanism by which elevated succinate levels can be harmful. *P. succinatutens* increases the gut barrier and alleviates the effect of succinate. However, despite its known role as a producer of short‐chain fatty acids, we did not observe a correlation between *Phascolarctobacterium* abundance and propionate levels in our clinical data, indicating that the underlying mechanism in vivo remains incompletely understood and may not be solely driven by SCFA production.

While we were able to validate our findings from two clinical cohorts in vitro, some limitations must be considered. The patient cohorts differed in mortality. Although the number of deceased patients was low in our discovery cohort, Cox regression with Firth's penalized likelihood was applied to account for this limitation [18], and the findings were further supported through the independent validation cohort, although the lack of power calculation for the size of the cohorts and the absence of comparator groups without liver disease or healthy controls limits interpretation. Decompensation within the 12‐month follow‐up period was not significantly associated with *Phascolarctobacterium*, and more detailed data as well as differing observation periods would be required to better assess its potential prognostic value. Additionally, the cell culture model we used is only suitable for short term cultures, as the cell polarization is not stable over a longer time, and *P. succinatutens* is an anaerobic bacterium. Furthermore, a limitation of this study is that the experimental design does not allow us to assess the specificity of the observed TEER effects for *Phascolarctobacterium*, as other commensal or pathogenic bacteria may likewise modulate epithelial barrier function. To overcome these limitations, prospective clinical trials including interventions to increase *Phascolarctobacterium* abundance are required.

## 5 Conclusion

Our study aimed to characterize the intestinal barrier function in patients with liver cirrhosis. We showed a connection between intestinal barrier dynamics and taxonomic composition by linking improving zonulin with an increased abundance of *Phascolarctobacterium*, which was associated with better outcomes. The association of *Phascolarctobacterium* abundance with improved barrier function, liver function, and increased survival probability was validated in an independent cohort. Lower abundance of succinate metabolizer *Phascolarctobacterium* was associated with higher levels of succinate, suggesting a mechanistic involvement. In vitro, we demonstrated that succinate impairs gut barrier function and that *P. succinatutens* alleviates the negative effects of succinate on the gut barrier. Our findings suggest that *Phascolarctobacterium* may be a candidate biomarker in cirrhosis for gut–liver axis assessment and may warrant further investigation as a potential next‐generation probiotic.

**Abbreviations**
ANCOM: Analysis of Composition of Microbiomes
AUROC: area under the receiver operating characteristic curve
BH: Benjamini–Hochberg
BMI: body mass index
CI: confidence interval
CO_2_
: carbon dioxide
DADA2: Divisive Amplicon Denoising Algorithm
DAO: diamine oxidase
DMEM: Dulbecco's Modified Eagle's Medium
DNA: deoxyribonucleic acid
ELISA: enzyme‐linked immunosorbent assay
HR: hazard ratio
IBD: inflammatory bowel disease
INR: international normalized ratio
LBP: lipopolysaccharide‐binding protein
LEfSe: Linear discriminant analysis Effect Size
MELD: Model for End‐Stage Liver Disease
NMR: nuclear magnetic resonance
orthoPLS‐DA: orthogonal Partial Least‐Squares Discriminant Analysis
OTU: operational taxonomic unit
p‐adj: adjusted p‐value
QIIME2: Quantitative Insights Into Microbial Ecology 2
sCD14: soluble CD14
SCFA: short‐chain fatty acid
TEER: transepithelial electrical resistance
VIP: variable importance in projection

## Author Contributions

**Rosa Haller:** methodology, formal analysis, investigation, writing – original draft, visualization. **Nicole Feldbacher:** investigation, resources, data curation, writing – review and editing. **Stefan Fürst:** data curation, writing – review and editing, **Johannes Woltsche:** data curation, writing – review and editing, **Lukas Gulden:** data curation, writing – review and editing, **Jakob Schwarzl:** data curation, writing – review and editing, **Julia Traub:** conceptualization, data curation, writing – review and editing, **Tobias Madl:** supervision, funding acquisition, writing – review and editing, **Hansjörg Habisch:** methodology, formal analysis, writing – review and editing, **Angela Horvath:** conceptualization, software, validation, visualization, writing – review and editing, **Vanessa Stadlbauer:** conceptualization, supervision, funding acquisition, writing – review and editing.

## Funding

This work was supported by the Austrian Science Fund (KLIF‐741 B34, KLI 741). R.H. is a PhD student in the doctoral program MOLMED from the Medical University of Graz. T.M. is grateful to the Austrian Science Fund (FWF) for excellence cluster 10.55776/COE14, Grants DOI 10.55776/P28854, 10.55776/I3792, 10.55776/DOC130, and 10.55776/W1226, the Austrian Research Promotion Agency (FFG) grants 864690 and 870454; the Integrative Metabolism Research Center Graz; the Austrian Infrastructure Program 2016/2017; the Styrian Government (Zukunftsfonds, doc.fund program); the City of Graz; and BioTechMed‐Graz (flagship project). This project was funded in part by the FFG (www.ffg.at) and the European Union (EFRE) under Grant 912192. For open access purposes, the author has applied a CC BY public copyright license to any author accepted manuscript version arising from this submission.

## Ethics Statement

Discovery cohort: Approved by the research ethics committee of the Medical University of Graz (23–096 ex 10/11), and performed according to the Declaration of Helsinki. Validation cohort: approved by the research ethics committee of the Medical University of Graz (29–280 ex 16/17), and performed according to the Declaration of Helsinki.

## Consent

All included patients provided written consent. Patients with disorders not allowing written consent were excluded after screening.

## Conflicts of Interest

The authors declare no conflicts of interest.

## Supporting information


Supporting Information S1

## Data Availability

Clinical data are available from the corresponding author upon reasonable request. 16S sequencing data are available in the NCBI Sequencing Read Archive (SRP132827, PRJNA933898).
