# Supplementary material for: Clinical and Mechanistic Association Between Intestinal Permeability and the Gut Microbiome in Cirrhosis: Role of Phascolarctobacterium
Source: United European Gastroenterol J. 2026 Jul 13;14(6):e70262. doi: 10.1002/ueg2.70262 (PMC13358567; doi:10.1002/ueg2.70262)
Supplement: Supplementary file 1 — Supporting Information S1 [file UEG2-14-e70262-s001.docx]

**Clinical and mechanistic association between intestinal permeability and the gut microbiome in cirrhosis: Role of *Phascolarctobacterium***

Rosa Haller^1,2^, Nicole Feldbacher,^1,2^, Stefan Fürst^1^, Johannes Woltsche^1^, Lukas Gulden^1^, Jakob Schwarzl^1^, Julia Traub^3^, Tobias Madl^4,5^, Hansjörg Habisch^4^, Angela Horvath^1,2^, Vanessa Stadlbauer^1,2,5^

^1^ Division of Gastroenterology and Hepatology, Department of Internal Medicine, Medical University of Graz, Graz, Austria

^2^ Center for Biomarker Research in Medicine (CBmed), Graz, Austria

^3^ Department of Clinical Medical Nutrition, University Hospital Graz, Graz, Austria

^4^Otto Loewi Research Center, Medicinal Chemistry, Medical University of Graz, Graz, Austria

^5^ BioTechMed-Graz, Graz, Austria

**Methods**

**Cell cultivation – Fibrinogen depletion of pHPL**

1M CaCl_2_ (C3881, Sigma Aldrich, St Louis, Missouri, USA) was added to a final concentration of 20mM to pHPL^1^. It was incubated at 37°C for one hour. Next, the pHPL was vortexed to reduce the size of the formed clots. The clots were removed by centrifugation (500xg, 10 minutes). The fibrinogen-depleted pHPL was stored at -20°C.

1. Kee LT, Lee YT, Ng CY, et al. Preparation of Fibrinogen-Depleted Human Platelet Lysate to Support Heparin-Free Expansion of Umbilical Cord-Derived Mesenchymal Stem Cells. *Biology (Basel)*. 2023;12(8):1085. doi:10.3390/biology12081085

**Bacteria cultivation – PY+X Medium with 8g/l sodium succinate (DSMZ Medium 104c)**

Table S1: Composition of DSMZ Medium 104c (PY+X) with 8g/l sodium succinate

| Trypticase peptone | 5 g | 70172, Sigma Aldrich, St Louis, Missouri, USA |
| --- | --- | --- |
| Meat peptone (pepsin-digested) | 5 g | 2366.3, Carl Roth, Karlsruhe, Germany |
| Yeast extract | 10 g | Y1625, Sigma Aldrich, St Louis, Missouri, USA |
| *Salt solution* | 40 ml |  |
| Sodium resazurin (0.1% w/v) | 0.5 ml | R7017, Sigma Aldrich, St Louis, Missouri, USA |
| L-Cysteine | 0.5 g | C1276, Sigma Aldrich, St Louis, Missouri, USA |
| Distilled water | 960 ml |  |
| *After autoclaving* | | |
| Sodium succinate | 8 g/l | 3195.2, Carl Roth, Karlsruhe, Germany |
| Na_2_CO_3_ | 1 g/l | S7795, Sigma Aldrich, St Louis, Missouri, USA |
| *Salt solution:* | | |
| CaCl_2_ x 2 H_2_O | 0.25 g | C3881, Sigma Aldrich, St Louis, Missouri, USA |
| MgSO_4_ | 0.5 g | M2773, Sigma Aldrich, St Louis, Missouri, USA |
| K_2_HPO_4_ | 1 g | P8281, Sigma Aldrich, St Louis, Missouri, USA |
| KH_2_PO_4_ | 1 g | P9791, Sigma Aldrich, St Louis, Missouri, USA |
| NaHCO_3_ | 10 g | 8551.1, Carl Roth, Karlsruhe, Germany |
| NaCl | 2 g | P029.2, Carl Roth, Karlsruhe, Germany |
| Distilled water | 1000 ml |  |

**Statistical analysis – R Packages**

Data processing

1. Wickham H, François R, Henry L, Müller K, Vaughan D (2023). _dplyr: A Grammar of Data Manipulation_. R package version 1.1.4, <https://CRAN.R-project.org/package=dplyr>.
2. Wickham H, Averick M, Bryan J, Chang W, McGowan LD, François R, Grolemund G, Hayes A, Henry L, Hester J, Kuhn M, Pedersen TL, Miller E, Bache SM, Müller K, Ooms J, Robinson D, Seidel DP, Spinu V, Takahashi K, Vaughan D, Wilke C, Woo K, Yutani H (2019). “Welcome to the tidyverse.” _Journal of Open Source Software_, *4*(43), 1686. doi:10.21105/joss.01686 <https://doi.org/10.21105/joss.01686>.
3. Ooms J (2024). _writexl: Export Data Frames to Excel 'xlsx' Format_. R package version 1.5.0, <https://CRAN.R-project.org/package=writexl>.
4. Wickham H, Bryan J (2023). _readxl: Read Excel Files_. R package version 1.4.3, <https://CRAN.R-project.org/package=readxl>.
5. Signorell A (2024). _DescTools: Tools for Descriptive Statistics_. R package version 0.99.55, <https://CRAN.R-project.org/package=DescTools>.
6. Kuhn M, Jackson S, Cimentada J (2022). _corrr: Correlations in R_. R package version 0.4.4, <https://CRAN.R-project.org/package=corrr>.
7. Long JA (2022). _jtools: Analysis and Presentation of Social Scientific Data_. R package version 2.2.0, <https://cran.r-project.org/package=jtools>.
8. Xavier Robin, Natacha Turck, Alexandre Hainard, Natalia Tiberti, Frédérique Lisacek, Jean-Charles Sanchez and Markus Müller (2011). pROC: an open-source package for R and S+ to analyze and compare ROC curves. BMC Bioinformatics, 12, p. 77. DOI: 10.1186/1471-2105-12-77 <http://www.biomedcentral.com/1471-2105/12/77/>
9. Therneau T (2024). _A Package for Survival Analysis in R_. R package version 3.7-0, <https://CRAN.R-project.org/package=survival>.
10. Kassambara A, Kosinski M, Biecek P (2024). _survminer: Drawing Survival Curves using 'ggplot2'_. R package version 0.5.0, <https://CRAN.R-project.org/package=survminer>.
11. R Core Team (2024). _R: A Language and Environment for Statistical Computing_. R Foundation for Statistical Computing, Vienna, Austria. <https://www.R-project.org/>.
12. Heinze G, Ploner M, Jiricka L, Steiner G (2023). _coxphf: Cox Regression with Firth's Penalized Likelihood_. R package version 1.13.4, <https://CRAN.R-project.org/package=coxphf>.
13. Heinze G, Ploner M, Jiricka L, Steiner G (2025). _logistf: Firth's Bias-Reduced Logistic Regression_. doi:10.32614/CRAN.package.logistf <https://doi.org/10.32614/CRAN.package.logistf>, R package version 1.26.1, <https://CRAN.R-project.org/package=logistf>.

Data visualization

1. H. Wickham. ggplot2: Elegant Graphics for Data Analysis. Springer-Verlag New York, 2016.
2. Kassambara A (2023). _ggpubr: 'ggplot2' Based Publication Ready Plots_. R package version 0.6.0, <https://CRAN.R-project.org/package=ggpubr>.
3. Ahlmann-Eltze, C., & Patil, I. (2021). ggsignif: R Package for Displaying Significance Brackets for 'ggplot2'. PsyArxiv. doi:10.31234/osf.io/7awm6
4. Nordmann, E., McAleer, P., Toivo, W., Paterson, H. & DeBruine, L. (2021). Data visualisation using R, for researchers who don't use R. Preprint.
5. van den Brand T (2024). _ggh4x: Hacks for 'ggplot2'_. R package version 0.2.8, <https://CRAN.R-project.org/package=ggh4x>.
6. Wilke C (2024). _cowplot: Streamlined Plot Theme and Plot Annotations for 'ggplot2'_. R package version 1.1.3, <https://CRAN.R-project.org/package=cowplot>.
7. Simon Garnier, Noam Ross, Robert Rudis, Antônio P. Camargo, Marco Sciaini, and Cédric Scherer (2024). viridis(Lite) - Colorblind-Friendly Color Maps for R. viridis package version 0.6.5.

Microbiome analysis

1. phyloseq: An R package for reproducible interactive analysis and graphics of microbiome census data. Paul J. McMurdie and Susan Holmes (2013) PLoS ONE 8(4):e61217.
2. Mikryukov V (2024). _metagMisc: Miscellaneous functions for metagenomic analysis_. R package version 0.5.0.
3. Oksanen J, Simpson G, Blanchet F, Kindt R, Legendre P, Minchin P, O'Hara R, Solymos P, Stevens M, Szoecs E, Wagner H, Barbour M, Bedward M, Bolker B, Borcard D, Carvalho G, Chirico M, De Caceres M, Durand S, Evangelista H, FitzJohn R, Friendly M, Furneaux B, Hannigan G, Hill M, Lahti L, McGlinn D, Ouellette M, Ribeiro Cunha E, Smith T, Stier A, Ter Braak C, Weedon J (2024). _vegan: Community Ecology Package_. R package version 2.6-8, <https://CRAN.R-project.org/package=vegan>.
4. Plantinga A, Chen J (2024). _pldist: Paired and Longitudinal Ecological Dissimilarities_. R package version 1.0.0.0000.
5. Yang Cao, Qingyang Dong, Dan Wang, Pengcheng Zhang, Ying Liu, Chao Niu. microbiomeMarker: an R/Bioconductor package for microbiome marker identification and visualization. Bioinformatics, 2022, btac438. doi: 10.1093/bioinformatics/btac438

**Results**

**Zonulin changes were associated with other gut-liver markers – Zonulin survival at baseline**


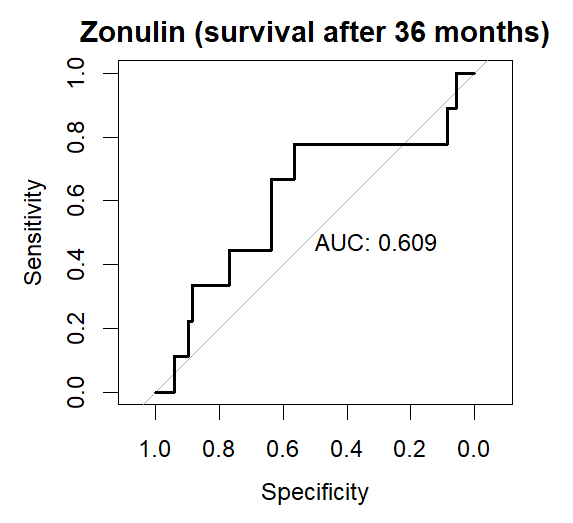


Figure S1: AUROC analysis of baseline stool zonulin levels for the prediction of 36-month survival.

**Zonulin changes were associated with other gut-liver markers – Development of medication, alcohol, decompensation and infections**

Table S2: Development of PPI intake, betablocker intake and alcohol cessation over six months, and new decompensation, severe and mild infections over twelve months in the discovery cohort.

| ***Discovery cohort*** | | | |
| --- | --- | --- | --- |
|  | ***Improved zonulin levels***  ***(n=33)*** | ***Deteriorating zonulin levels (n=45)*** | ***p-value*** |
| ***Six-month changes*** | | |  |
| **PPI stop** | 2 | 2 | 0.90 |
| **PPI start** | 1 | 3 |  |
| **Betablocker stop** | 0 | 0 | 0.86 |
| **Betablocker start** | 2 | 3 |  |
| **Alcohol cessation** | 3 | 4 | 0.80 |
| ***12-month changes*** | | |  |
| **New Decompensation** | 9 | 10 | 0.81 |
| **Severe Infections** | 2 | 5 | 0.69 |
| **Mild infections** | 1 | 3 | 0.63 |


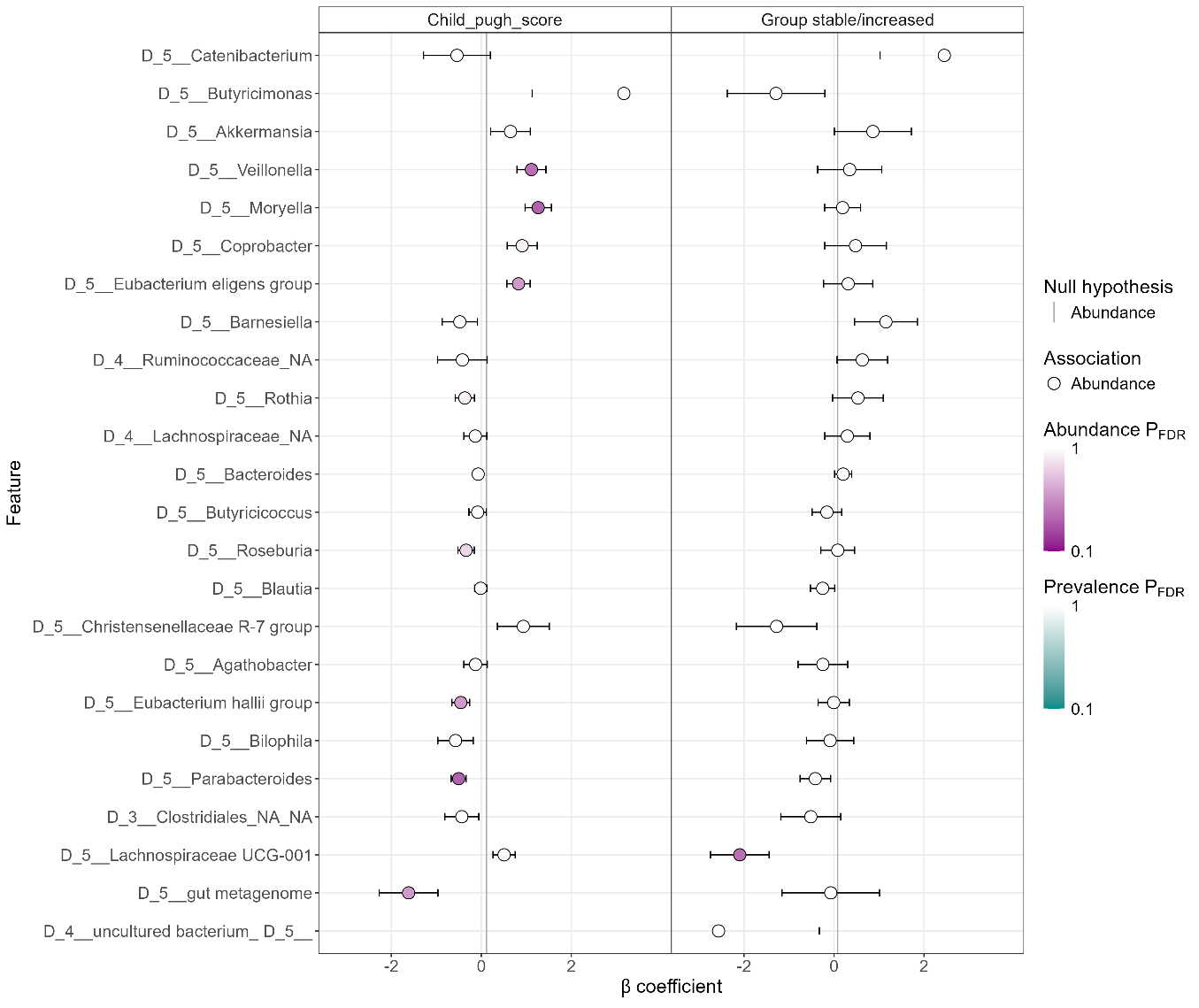


Figure S2: MaAsLin 3 did not identify any significant features depending on zonulin development.

***Phascolarctobacterium* was associated with a better prognosis – Influence of Phascolarctobacterium on survival in the validation cohort**


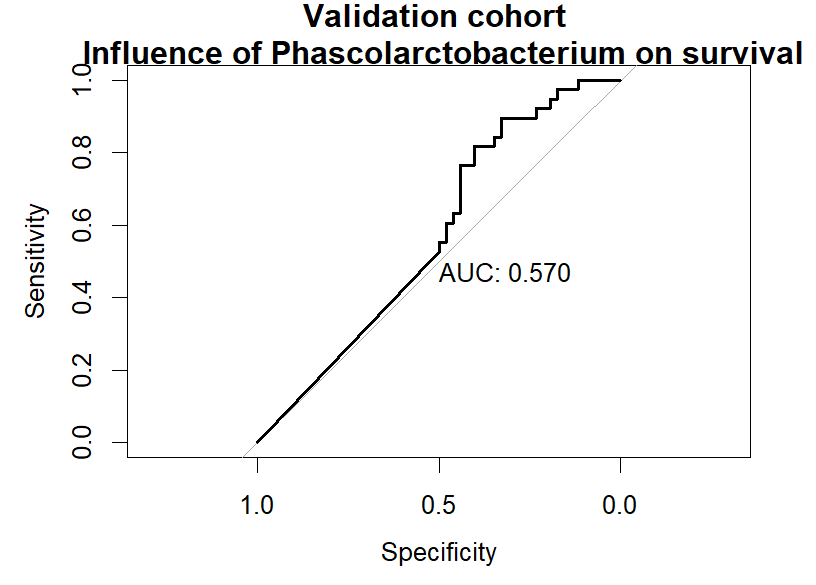


Figure S3: AUROC of *Phascolarctobacterium* abundance and mortality after 36 months in the validation cohort.

**Succinate impairs gut barrier function and Phascolarctobacterium improves gut barrier function in a cell culture model – TEER values**

Table S3: TEER values and difference.

| **Succinate treatment** | | | | | | | | | |
| --- | --- | --- | --- | --- | --- | --- | --- | --- | --- |
| **0mM** | **50mM** | | **100mM** | | **110mM** | | **120mM** | | **130mM** |
| *Baseline* | | | | | | | | | |
| 1628 | 1632 | | 1795 | | 1732 | | 1666 | | 1873 |
| 1743 | 1636 | | 1673 | | 1606 | | 1617 | | 1649 |
| *17 hours* | | | | | | | | | |
| 1704 | 1191 | | 1279 | | 1277 | | 1312 | | 1422 |
| 1604 | 1198 | | 1476 | | 1296 | | 1015 | | 1335 |
| *Difference* | | | | | | | | | |
| 76 | -442 | | -516 | | -455 | | -354 | | -451 |
| -139 | -438 | | -196 | | -310 | | -603 | | -315 |
| *Difference in percent* | | | | | | | | | |
| 5 | -27 | | -29 | | -26 | | -21 | | -24 |
| -8 | -27 | | -12 | | -19 | | -37 | | -19 |
| *Baseline* | | | | | | | | | |
| 1681 | 1810 | | 1633 | | 1583 | | 1823 | | 2050 |
| 1663 | 1541 | | 1671 | | 1268 | | 1250 | | 1936 |
| *17 hours* | | | | | | | | | |
| 1681 | 1805 | | 1915 | | 1630 | | 1569 | | 932 |
| 1881 | 1685 | | 1832 | | 1576 | | 2024 | | 1231 |
| *Difference* | | | | | | | | | |
| 1 | -5 | | 282 | | 47 | | -254 | | -1118 |
| 219 | 144 | | 161 | | 308 | | 774 | | -705 |
| *Difference in percent* | | | | | | | | | |
| 0 | 0 | | 17 | | 3 | | -14 | | -55 |
| 13 | 9 | | 10 | | 24 | | 62 | | -36 |
| *Baseline* | | | | | | | | | |
| 1847 | 1993 | | 2119 | | 1998 | | 1985 | | 1862 |
| 1973 | 2439 | | 2151 | | 2229 | | 1885 | | 1950 |
| *17 hours* | | | | | | | | | |
| 1929 | 2095 | | 1980 | | 1775 | | 493 | | 175 |
| 2004 | 2401 | | 1934 | | 1374 | | 637 | | 121 |
| *Difference* | | | | | | | | | |
| 82 | 102 | | -139 | | -224 | | -1491 | | -1687 |
| 31 | -38 | | -217 | | -854 | | -1248 | | -1828 |
| *Difference in percent* | | | | | | | | | |
| 4 | 5 | | -7 | | -11 | | -75 | | -91 |
| 2 | -2 | | -10 | | -38 | | -66 | | -94 |
| **140mM** | | **150mM** | | **160mM** | | **170mM** | | **200mM** | |
| *Baseline* | | | | | | | | | |
| 1497 | | 1903 | | 1803 | | 1605 | | 1467 | |
| 1803 | | 1845 | | 1873 | | 1606 | | 1798 | |
| *17 hours* | | | | | | | | | |
| 1174 | | 1362 | | 615 | | 91 | | 8 | |
| 1292 | | 1317 | | 228 | | 482 | | -3 | |
| *Difference* | | | | | | | | | |
| -322 | | -541 | | -1188 | | -1514 | | -1459 | |
| -511 | | -528 | | -1645 | | -1124 | | -1801 | |
| *Difference in percent* | | | | | | | | | |
| -22 | | -28 | | -66 | | -94 | | -99 | |
| -28 | | -29 | | -88 | | -70 | | -100 | |
| *Baseline* | | | | | | | | | |
| 1674 | | 1602 | | 1833 | | 1504 | | 1662 | |
| 1593 | | 1918 | | 1678 | | 1804 | | 1540 | |
| *17 hours* | | | | | | | | | |
| 522 | | 76 | | 9 | | 43 | | -1 | |
| 994 | | 165 | | 59 | | 26 | | 19 | |
| *Difference* | | | | | | | | | |
| -1153 | | -1527 | | -1824 | | -1460 | | -1663 | |
| -599 | | -1753 | | -1619 | | -1779 | | -1521 | |
| *Difference in percent* | | | | | | | | | |
| -69 | | -95 | | -99 | | -97 | | -100 | |
| -38 | | -91 | | -96 | | -99 | | -99 | |
| *Baseline* | | | | | | | | | |
| 2004 | | 2041 | | 1757 | | 2147 | | 2083 | |
| 1987 | | 2072 | | 2173 | | 1984 | | 1938 | |
| *17 hours* | | | | | | | | | |
| 103 | | -5 | | -5 | | -4 | | -2 | |
| 14 | | 10 | | -5 | | -9 | | -9 | |
| *Difference* | | | | | | | | | |
| -1901 | | -2045 | | -1762 | | -2151 | | -2084 | |
| -1973 | | -2062 | | -2177 | | -1993 | | -1946 | |
| *Difference in percent* | | | | | | | | | |
| -95 | | -100 | | -100 | | -100 | | -100 | |
| -99 | | -99 | | -100 | | -100 | | -100 | |

| ***Phascolarctobacterium* treatment** | | | | | | | |
| --- | --- | --- | --- | --- | --- | --- | --- |
| **Control** | **140mM Succinate** | | **130mM Succinate** | | ***P. succinatutens* B162** | | ***P. succinatutens A177*** |
| *Baseline* | | | | | | | |
| 1781 | 1957 | | 1717 | | 1925 | | 1786 |
| 2006 | 2165 | | 1975 | | 1779 | | 1601 |
| *17 hours* | | | | | | | |
| 1313 | 490 | | 166 | | 2710 | | 2618 |
| 1239 | 514 | | 278 | | 2560 | | 2506 |
| *Difference* | | | | | | | |
| -468 | -1466 | | -1551 | | 785 | | 831 |
| -767 | -1651 | | -1698 | | 781 | | 905 |
| *Difference in percent* | | | | | | | |
| -26 | -75 | | -90 | | 41 | | 47 |
| -38 | -76 | | -86 | | 44 | | 57 |
| *Baseline* | | | | | | | |
| 1808 | 1925 | | 1679 | | 1879 | | 1799 |
| 1882 | 1603 | | 1767 | | 1712 | | 1916 |
| *17 hours* | | | | | | | |
| 1614 | 268 | | 1112 | | 3294 | | 3064 |
| 1702 | 695 | | 1127 | | 3134 | | 3226 |
| *Difference* | | | | | | | |
| -194 | -1657 | | -567 | | 1415 | | 1265 |
| -180 | -908 | | -640 | | 1422 | | 1311 |
| *Difference in percent* | | | | | | | |
| -11 | -86 | | -34 | | 75 | | 70 |
| -10 | -57 | | -36 | | 83 | | 68 |
| *Baseline* | | | | | | | |
| 1849 | 1804 | | 1704 | | 1862 | | 1907 |
| 1850 | 1669 | | 1918 | | 1962 | | 1778 |
| *17 hours* | | | | | | | |
| 1539 | 65 | | 664 | | 3282 | | 2964 |
| 1553 | 94 | | 224 | | 3095 | | 2967 |
| *Difference* | | | | | | | |
| -310 | -1739 | | -1040 | | 1420 | | 1056 |
| -298 | -1575 | | -1694 | | 1133 | | 1189 |
| *Difference in percent* | | | | | | | |
| -17 | -96 | | -61 | | 76 | | 55 |
| -16 | -94 | | -88 | | 58 | | 67 |
| **140mM Succinate + *P. succinatutens* B162** | | **130mM Succinate+ *P. succinatutens* B162** | | **140mM Succinate +*P. succinatutens A177*** | | **130mM Succinate +*P. succinatutens A177*** | |
| *Baseline* | | | | | | | |
| 1873 | | 1888 | | 1604 | | 1807 | |
| 1913 | | 1839 | | 1787 | | 1764 | |
| *17 hours* | | | | | | | |
| 2051 | | 2000 | | 1793 | | 2234 | |
| 2303 | | 2029 | | 2166 | | 2097 | |
| *Difference* | | | | | | | |
| 179 | | 112 | | 189 | | 427 | |
| 391 | | 190 | | 379 | | 333 | |
| *Difference in percent* | | | | | | | |
| 10 | | 6 | | 12 | | 24 | |
| 20 | | 10 | | 21 | | 19 | |
| *Baseline* | | | | | | | |
| 1913 | | 1712 | | 1642 | | 1842 | |
| 1877 | | 1845 | | 1846 | | 1771 | |
| *17 hours* | | | | | | | |
| 1979 | | 2154 | | 1305 | | 1581 | |
| 2304 | | 2252 | | 1672 | | 1276 | |
| *Difference* | | | | | | | |
| 65 | | 441 | | -337 | | -261 | |
| 427 | | 407 | | -174 | | -495 | |
| *Difference in percent* | | | | | | | |
| 3 | | 26 | | -21 | | -14 | |
| 23 | | 22 | | -9 | | -28 | |
| *Baseline* | | | | | | | |
| 1960 | | 1988 | | 2038 | | 1890 | |
| 2026 | | 1882 | | 1970 | | 1868 | |
| *17 hours* | | | | | | | |
| 1419 | | 1298 | | 1508 | | 1711 | |
| 1355 | | 934 | | 1180 | | 1595 | |
| *Difference* | | | | | | | |
| -541 | | -690 | | -531 | | -180 | |
| -671 | | -948 | | -790 | | -273 | |
| *Difference in percent* | | | | | | | |
| -28 | | -35 | | -26 | | -10 | |
| -33 | | -50 | | -40 | | -15 | |
